# Methimazole-induced congenital hypothyroidism affects gonocytes differentiation and arrests meiosis: role of Sertoli cells

**DOI:** 10.3389/fcell.2024.1493872

**Published:** 2024-11-21

**Authors:** Andrea Gómez-Zúñiga, Daniel Adrián Landero-Huerta, Julio César Rojas-Castañeda, Karla Sánchez-Huerta, Itzel Jatziri Contreras-García, Rafael Reynoso-Robles, Marcela Arteaga-Silva, Rosa María Vigueras-Villaseñor

**Affiliations:** ^1^ Posgrado en Biología Experimental, Universidad Autónoma Metropolitana Unidad Iztapalapa, Mexico City, Mexico; ^2^ Laboratorio de Biología de la Reproducción, Instituto Nacional de Pediatría, Mexico City, Mexico; ^3^ Laboratorio de Neurociencias, Instituto Nacional de Pediatría, Mexico City, Mexico; ^4^ Laboratorio de Morfología Celular y Tisular, Instituto Nacional de Pediatría, Mexico City, Mexico; ^5^ Laboratorio de Neuroendocrinología Reproductiva, División de Ciencias Biológicas y de la Salud, Departamento de Biología de la Reproducción, Universidad Autónoma Metropolitana Iztapalapa, Mexico City, Mexico

**Keywords:** congenital hypothyroidism, Sertoli cells, gonocyte, meiosis, methimazole

## Abstract

**Background:**

Congenital hypothyroidism (CH) is a pathology that affects various organs, including the testicles. The mechanisms by which this condition alters fertility is unknown. This study aimed at determining if experimental CH affects gonocyte differentiation and arrests meiosis; and the possible role of the Sertoli cell (SC) in this condition.

**Material and Methods:**

Two groups of rats consisting of Control group and Methimazole (MMI) induced CH rats were formed. The induction of CH was achieved by the administration of MMI starting on day 16 postcoitum and continued until euthanized. Euthanasia was performed at 5, 8, 10, 16 and 64 days of age. Following this, the testicular tissue of each animal was extracted and processed for histopathological and ultrastructural analysis. In addition, the tissue was used for the determination of proteins and their transcriptions, events which are characteristics of gonocyte differentiation. The SC functionality proteins was determined immunohistochemically, while sperm parameters of the cauda epididymis were verified.

**Results:**

CH caused a delay in the gonocyte differentiation, and arrested meiosis and spermiogenesis. These events had long-term repercussions on the quality of the seminiferous epithelium. The results show that CH induces alterations in the functional state of SCs that may have led to the deficiency in the synthesis and/or in the release of molecules necessary for gonocyte differentiation; as well as disorders in the process of meiosis that resulted in sperm absence.

**Conclusion:**

These results suggest that CH affects gonocyte differentiation and arrests meiosis, possibly through altering the functional status of SCs.

## 1 Introduction

Congenital hypothyroidism (CH) is one of the most common endocrine disorders (Grüters and Krude, 2011). Its prevalence in Europe is put to one in every 2,000 to 3,000 newborns (van Trotsenburg et al., 2021). It is a pathology of great importance for its effects on metabolism (Pan et al., 2019); and on different organs, such as the skin, heart, kidneys, among others (Maciel et al., 2013; Yousefichaijan et al., 2015; Omidi et al., 2020). Induced CH in experimental rodents has also been associated with fertility problems as a result of decreased concentrations of circulating testosterone, progesterone, prolactin, and negative effect on sperm quality or inhibition of spermatogenesis (Umezu et al., 2004; Anbalagan et al., 2010; Aiceles et al., 2017).

It is known that thyroid hormones (TH) are necessary for the maturation of Sertoli cells (SCs) (Palmero et al., 1989; Bunick et al., 1994; de França et al., 1995), as well as for their ultrastructural, biochemical and molecular maintenance. Hence, they are essential for adequate spermatogenesis (Cooke et al., 1994; de França et al., 1995; Palmero et al., 1995; Zanatta et al., 2013). It has been reported that in rdw rats with CH due to missense mutation of the thyroglobulin gene, the SCs are unable to detain their proliferations, which is a necessary process for the maturation of these cells (Sakai et al., 2004). In the rat, the receptor for TH (TR) α1 (TRα1) isoform is the one expressed in immature, proliferating and adult SCs. In turn, TRα and TR β1 isoforms are expressed in spermatogonia, spermatocytes, round and elongated spermatids (Buzzard et al., 2000; Fadlalla et al., 2017).

In addition, the expression of TRs, in rats, is high at birth with a peak at 16 days *postpartum* (dpp) and decreases in adulthood (Buzzard et al., 2000; Jannini et al., 2000). During this period of maximum expression**,** gonocytes differentiate into spermatogonia, and the first spermatogenic wave begins with meiosis (Culty, 2009).

Gonocytes synthesize proteins as POU domain class 5 transcription factor 1 (POU5F1), (Zhao and Garbers, 2002); transcription factor AP2 (TFAP2C) (Chazaud et al., 1996); sal-like protein 4 (SALL4) (Eildermann et al., 2012); that confer to them pluripotent ability, undifferentiated characteristic, and survival potential. During their differentiation, these proteins are downregulated and the cells migrate towards the basal membrane where they change shape and begin the expression of Stra8 protein, events that are purely characteristics of spermatogonia (Culty, 2009). The Stra8 protein is necessary for the initiation and continuity of meiosis (Koubova et al., 2006). Simorangkir et al. (1997) reported a delay in the differentiation of gonocytes in experimental neonatal hypothyroidism induced by the administration of propylthiouracil (PTU).

Therefore, the mechanisms of damage generated by CH on gonocyte differentiation, the inhibition of spermatogenesis and sperm quality previously reported are not known (Umezu et al., 2004; Anbalagan et al., 2010; Aiceles et al., 2017). Hence, the evidences here exposed, depict the possibility that TH regulate the function of SCs for the production of paracrine molecules that are necessary in the differentiation of gonocytes and in the initiation and continuation of meiosis (Cooke et al., 1994; de França et al., 1995; Palmero et al., 1995; Simorangkir et al., 1997; Zanatta et al., 2013; Meroni et al., 2019). Therefore, the objective of the present work was to determine the mechanisms by which experimental CH affects gonocyte differentiation and stops germ cell meiosis, as well as the role of the SC in this affectation.

## 2 Material and methods

### 2.1 Animals, experimental design, and sample collection

The animals were handled in accordance with the official Mexican standard (SAGARPA NOM-062-Z00-1999) “Technical specifications for the production, care and use of laboratory animals” and with the internal regulation on ethical principles and treatment of animals of the Institutional Committee for the Care and Use of Laboratory Animals of the National Institute of Pediatrics, Mexico City, Mexico, with registration number INP019/2019.

Wistar rats were bred and maintained in the vivarium of the National Institute of Pediatrics. The animals were allowed free access to food (Rodent Lab Chow 5001, Purina Inc.) and water, and maintained at a temperature of 21°C ± 2°C, relative humidity 50% ± 10% and a 12-h light/dark cycles (light on 06:00). At night, the male rats (∼250 g body weight) were paired with females (∼200 g body weight) in estrus to have pups. The next morning, vaginal smears were obtained from the females to confirm the presence of sperm. If the vaginal smear was positive, this day was recorded as day 0 postcoitum. Subsequently, the pregnant animals were divided into two groups: the Control (without administration of any drug in the drinking water) and the exposed to methimazole (MMI). The administration of MMI (1-methyl-3H-imidazole-2-thione Sigma-Aldrich, Mexico City, Mexico) was via drinking water containing the substance (23 mg/kg body weight/day) administered to the mothers from 16 days postcoitum (dpc) until the pups were euthanized. This dose was calculated daily based on water consumption the day before its preparation. In rats, the formation of thyroid gland is completed on the day 16 of gestation, and the synthesis of TH is begun. MMI acts by inhibiting the activity of thyroid peroxidase, which participates in the iodination of tyrosine residues, preventing the synthesis of TH (Awosika et al., 2023).

**FIGURE 1:**
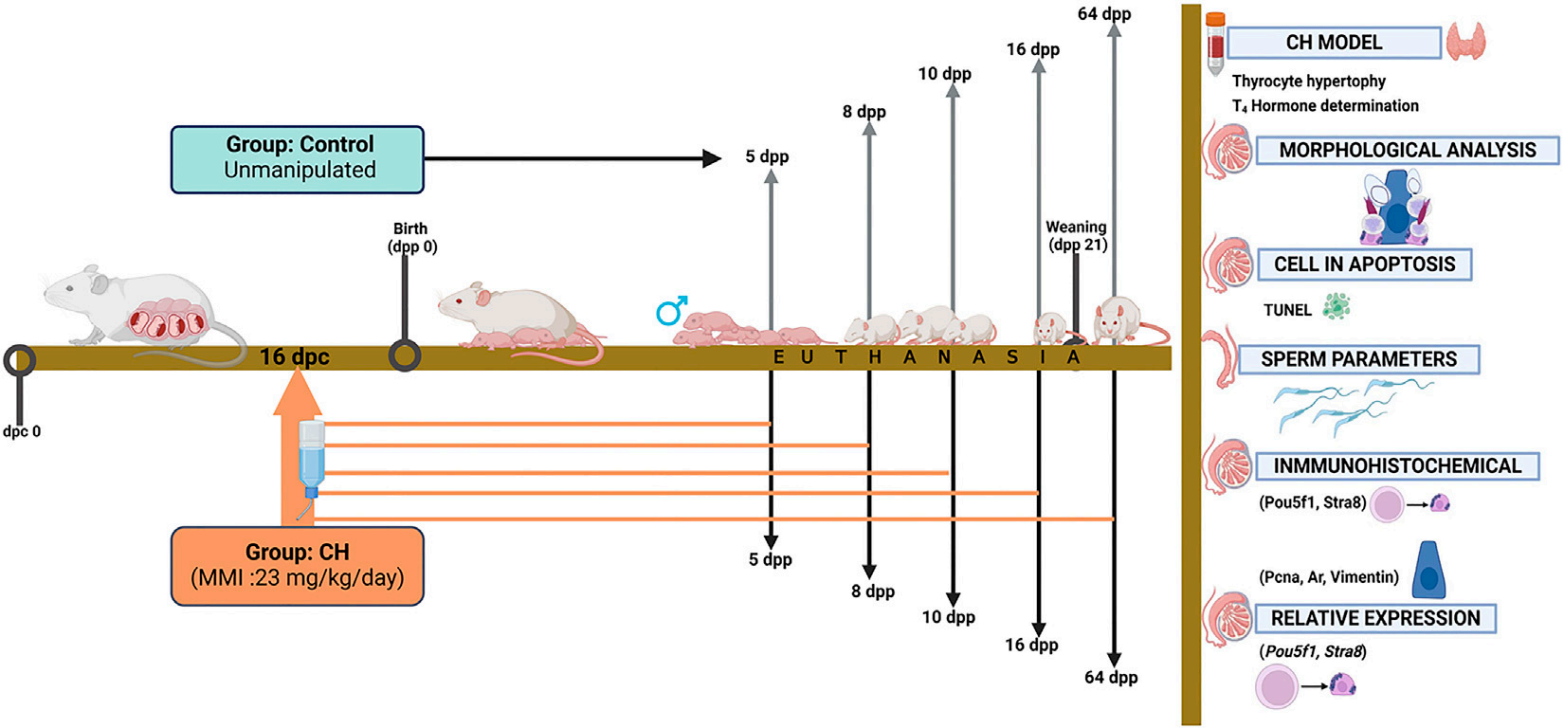
Experimental design and sample processing. The Control group received no drug in the drinking water and the CH group received MMI from 16 dpc. Euthanasia was carried out in both groups at 5, 8, 10, 16 and 64 dpp. Blood and thyroid samples were obtained for model validation. The testes were analyzed for morphology, ultrastructure; and apoptosis and sperm parameters were determined. Specific proteins were analyzed by immunohistochemistry and relative expression to determine gonocyte differentiation and Sertoli cell functionality. CH, congenital hypothyroidism; MMI, methimazole; dpc, days postcoitum; dpp, days *postpartum*.

At birth, the litters were adjusted to 8 pups and all the males were maintained and kept in the vivarium until euthanized on 5, 8, 10, 16 and 64 dpp. The sacrifice of the animals was carried out by an overdose of pentobarbital administered intraperitoneally in saline solution (120–150 mg/kg) after sedation with xylazine (10 mg/kg) (Pfizer, Mexico City, Mexico). Also, the body and testicular weight of each animal was recorded (Figure 1).

### 2.2 Thyroxine (T_4_) hormone determination

The determination of T_4_ concentrations was performed to verify the presence of CH in the animals. For this, blood sample was obtained by cardiac puncture from each of the animals between 1:00 p.m. and 2:00 p.m. to avoid circadian fluctuations. The samples were centrifuged at 12,000 revolutions per minute for 10 min to obtain serum. T_4_ determination was performed using an ELISA kit (Calbiotech, Cordell Court, El Cajon, CA) following the manufacturer’s instructions.

### 2.3 Morphological analysis

Half of the right testicular sample plus the thyroid gland tissue were fixed in Karnovsky solution for 2 h. Later, they were postfixed in 1% osmium tetroxide (OsO_4_) (Sigma Aldrich, St Louis MO. United States), dehydrated and processed for inclusion in Epon 812 (Ted Pella, Inc., Redding, CA, United States). Sectioning of the samples were made at 1 µm thickness using an ultramicrotome (Ultracut UCT, Leica, Vienna, Austria) and were stained with 0.5% toluidine blue. Histological analysis was performed using a BX 51 Olympus optical microscope (Olympus Corp., Tokyo, Japan). Fifteen to twenty cross sections of the seminiferous cords/tubes were evaluated for each animal. The area of the seminiferous epithelium was determined using an image analysis system (Image-Pro Plus 7.0, Media Cybernetics, INC. MD, United States). The number of Sertoli cells, gonocytes, spermatogonia, spermatocytes, round and elongated spermatids per cords/tubes seminiferous was obtained. The degree of maturation of the seminiferous epithelium (MI), called the Johnsen Index (Johnsen, 1970) and the histopathological index (HI) (Vigueras-Villaseñor et al., 2009) were also determined.

The gonocyte differentiation index (number of spermatogonia/number of gonocytes) was obtained. Finally, the thyroid gland was analyzed to determine thyrocyte hypertrophy in the CH model. All histological evaluations were performed by a single observer.

### 2.4 Determination of cells in apoptosis

To determine apoptosis, the terminal deoxynucleotidyl transferase-mediated dUDP nick-end labelling (TUNEL) assay was used. The testicular portions were fixed in 4% paraformaldehyde for 18 h. These tissue samples were dehydrated, classified and embedded in paraffin. Afterwards, each tissue sample was cut at 4 μm thickness and mounted on a slide coated with poly-L-lysine (Sigma Aldrich). Subsequently, it was deparaffinized and dehydrated in a graded series of alcohols. Then, the tissue sections were treated with Triton X-100 solution (Sigma) at 0.1% for 2 min. They were later incubated with TUNEL solution (50 µL terminal deoxynucleotidyl transferase and 450 µL nucleotide mixture, *in situ* Cell Death Detection Kit, Roche Diagnostic Corporation, Indianapolis, IN, United States) for 1 h at 37°C. The tissue sections were then mounted with fluorescence medium (Fluoroshield Mounting Medium with DAPI, ABCAM) for observation using an Olympus fluorescence microscope (Olympus BX51). The number of apoptotic cells per seminiferous cords/tubes was calculated and the results were expressed as the number of apoptotic cells per cross section. 15 to 20 transverse sections of the seminiferous cords/tubes were evaluated for each of the animals. All dilutions and washes between steps were performed using PBS (0.1 M). Negative control sections were processed identically, but the enzyme solution (terminal deoxynucleotidyl transferase) was omitted. All histological examinations were performed by a single observer.

### 2.5 Sperm parameters

The epididymis of each rat was dissected, and the adjacent associated tissue was removed, and placed in saline (154 mM NaCl at 38°C). The caudal portion of the epididymis was sectioned by radial cuts using fine-point scissors. This section was incubated for 5 min in 2 mL of BTS (Beltsville Thawing Solution, BTS™, Minitub™, Hauptstrasse, Tiefenbach, Germany) with occasional gentle shakes to allow the release of sperm into the medium. Subsequently, 10 µL of the medium with sperm were taken and placed on slides that were stained with hematoxylin-eosin to determine the percentage of normal sperm, discarding morpho-abnormalities (alterations in the head, midpiece, tail) per 100 sperm (Vigueras-Villaseñor et al., 2011). Sperm concentration was determined in a Neubauer chamber (BRANDGMBH, Wertheim, Germany) using a solution diluted in saline solution (1:20), and the results were expressed x10^6^/mL. The percentage of vitality was obtained by evaluating 100 spermatozoa stained with Coomassie brilliant blue (BBC) (0.22% Coomassie blue G-250, in 50% methanol, 10% glacial acetic acid and 40% distilled water). All the analysis were performed in triplicate with Olympus BX51 microscope.

### 2.6 Immunohistochemical analysis

Tissue samples from the half of the right testicular of each of the animals were fixed in 4% paraformaldehyde (Sigma-Aldrich) and processed for inclusion in paraffin. Sections of 4 μm thickness were made and mounted on slides covered with poly-L-lysine (Sigma-Aldrich, St. Louis, MO, United States). Tissue sections were deparaffinized with xylene and hydrated with graded ethanol solutions. They were subsequently incubated with citrate buffer (0.01M, pH 7.6) for 5 min in a microwave oven at 800 W. Then, the sections were washed three times each for 5 min with phosphate buffer (PBS)/0.1% Tween 20 (Sigma-Aldrich) and blocked with 1% bovine serum albumin in PBS for 2 h in a humid chamber. Subsequently, they were incubated overnight at room temperature with the corresponding antibody. The following mouse monoclonal primary antibodies were used: 1) Anti-Pou5f1 antibody, pluripotency protein used to identify gonocytes (sc-5279, Santa Cruz, Biotechnology, CA, United States), at a 1:25 dilution; 2) Anti-proliferating-cell nuclear antigen (Pcna) antibody, Sertoli cell proliferation protein (sc-56, Santa Cruz, Biotechnology) at a 1:50 dilution; 3) Anti-Vimentin antibody, structural protein that allows the determination of the functionality of Sertoli cells, (sc-6260, Santa Cruz Biotechnology) at a 1:30 dilution. In addition, rabbit polyclonal primary antibodies were used as follows: 1) Anti-Stra8 antibody, protein for the initiation of meiosis, characteristic of spermatogonia (ab49602, ABCAM, Cambridge, United Kingdom) at a 1:50 dilution and 2) Anti-Androgen Receptor (Ar) antibody, a protein that allows the determination of the functionality of Sertoli cells (sc-816, Santa Cruz Biotechnology) at a 1:30 dilution. This was followed by another incubation with biotinylated secondary antibodies (Horse anti-mouse IgG Biotinylated BA 2000, lot ZJ1212 Vectastain Vector Labs, Burlington, ON, Canada, and with Goat anti-rabbit IgG Biotinylated, cat BA1000, Lot ZE1218, Vectastain Vector, respectively), at a dilution of 1:200 for 2 h at room temperature. The samples were then exposed to Avidin-Biotin System conjugate (ABC kit, Vectastain Vector Labs) for 1 h at room temperature. The reaction was evidenced using diaminobenzidine (DAB Chromogen Kit 901-DB801-032118, Biocare, Concord, CA, United States). Subsequently, tissue sections were counterstained with Gill’s hematoxylin, and coverslipped with entellan. The number of immunoreactive cells per transversely cut seminiferous cords/tubes was quantified. 15 to 20 seminiferous tubes per animal were evaluated. The distribution pattern of vimentin immunoreactivity in the cytoplasm of SCs was also determined.

### 2.7 Determination of the relative expression of *Stra8* and *Pou5f1*

For the animals of 5–16 dpp, total RNA was extracted from the tissue obtained from the halves of both testes of each animal, while for the animals of 64 dpp, the extraction of total RNA was from the testicular tissue obtained from the central part of each testis using the Qiagen^®^ RNeasy Lipid Tissue Mini Kit (Cat. 74804, Hilden, Germany). Subsequently, relative expression was determined by RT-qPCR assays. For reverse transcription, the TaqMan Reverse Transcription Reagents kit (Life Technologies, United States) was used to obtain cDNA from aliquots of 100 ng of RNA for each sample. Subsequently, qPCR assays were performed using the TaqMan Universal Master Mix kit (Thermo Fisher, UK). 5 μL of Taqman Universal PCR Master Mix, 0.5 µL of the test probe as appropriate, *Stra8* (RN01747849_m1) and *Pou5f1* (Rn06413993_s1), 3.5 µL of nuclease-free water and 1 µL of cDNA were used for a total volume of 10 µL. Reactions were performed in triplicate in a thermocycler (Applied Biosystem Step One TM, Foster City, CA, United States) under the following conditions: 50°C for 2 min, 95°C for 10 min, 40 cycles at 95°C for 15 s and 60°C for 1 min. Expression levels were determined using the 2^−ΔΔCT^ method. For data normalization, the probe (*Gapdh*, RN01775763_g1) was used as an endogenous control.

### 2.8 Statistical analysis

All data were expressed as the mean ± SEM. The Student’s T-test was used for statistical analysis in the SPSS package and values *p* < 0.05 was considered significant.

## 3 Results

### 3.1 Congenital hypothyroidism model

Animals with CH showed a significant reduction in body and testicular weight at dpp 16 and 64 compared to the Control, as well as in T_4_ concentrations (Table 1, *p* < 0.05), with thyrocyte hypertrophy (complementary Figure).

**TABLE 1:** Parameters evaluated in Control and CH animals.

Age (dpp)	Group	[T_4_] (μg/dL)	Bw (g)	Tw (g x10^−2^)	g/cord	Sg/cord/tube	DI
**5**	*CONTROL*	3.46 ± 1.41	11.60 ± 0.89	1.00 ± 0.10	1.86 ± 0.24	-	-
	*CH*	0.12 ± 0.02*	10.20 ± 1.30	0.9 ± 0.06	2.21 ± 0.20*	-	-
**8**	*CONTROL*	4.24 ± 0.16	15.60 ± 3.60	1.80 ± 0.10	1.33 ± 0.41	2.30 ± 0.74	1.84 ± 0.64
	*CH*	0.98 ± 0.23*	18.40 ± 0.50	1.60 ± 0.03	1.88 ± 0.62*	0.77 ± 0.56*	0.36 ± 0.18*
**10**	*CONTROL*	8.02 ± 1.25	23.40 ± 1.10	2.70 ± 0.20	1.57 ± 0.46	16.15 ± 6.31	12.65 ± 8.92
	*CH*	0.17 ± 0.02*	22.50 ± 2.40	2.00 ± 0.30	2.73 ± 0.46*	4.57 ± 1.56*	1.64 ± 0.37*
**16**	*CONTROL*	7.98 ± 1.80	28.83 ± 0.70	15.00 ± 1.50	-	16.89 ± 3.50	-
	*CH*	1.76 ± 0.94*	20.20 ± 1.40*	3.00 ± 0.30*	-	9.05 ± 2.78*	-
**64**	*CONTROL*	8.10 ± 0.93	311.20 ± 6.60	161.00 ± 6.00	-	21.15 ± 1.69	-
	*CH*	0.24 ± 0.06*	79.60 ± 6.40*	86.00 ± 23.00*	**-**	13.03 ± 4.36*	-

Data are expressed as mean ± SEM, of 5 animals/group. Analyzing 15 to 20 seminiferous cords/tubes per animal. Statistically significant difference compared to the Control group = *p* < 0.05. dpp: days *postpartum*, CH: congenital hypothyroidism, Bw: Body weight, Tw: testicular weight, g: gonocyte, Sg: Spermatogonia, DI: Differentiation index.

### 3.2 Gonocytes differentiation and meiosis arrest

#### 3.2.1 Morphological analysis

**FIGURE 2:**
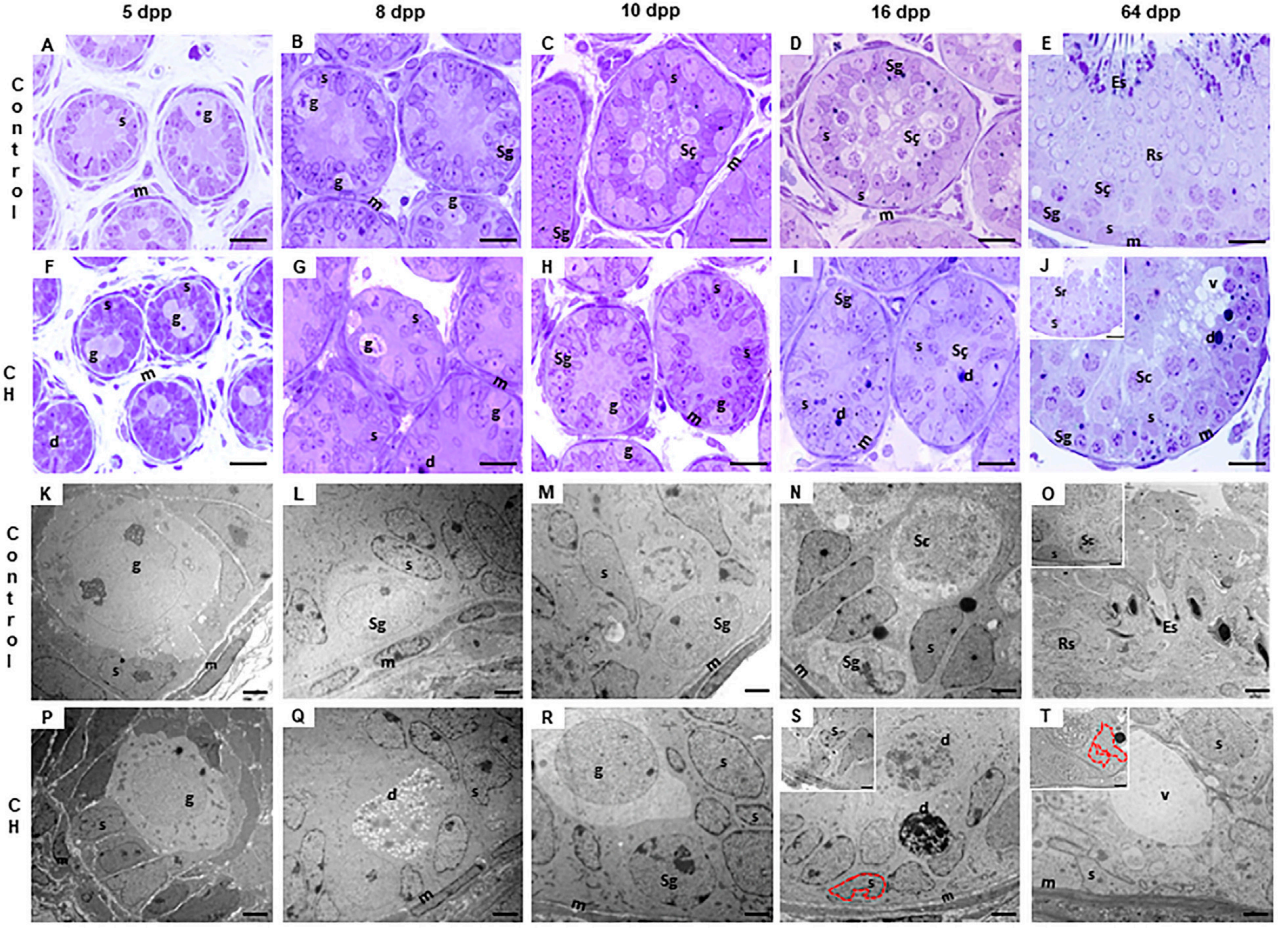
Cords/tubes seminiferous of different ages. It is observed in the group with CH **(F–J, P–T)**, unlike the Control **(A–E, K–O)**, the permanence of gonocytes until 10 dpp **(H)**; delay in the emergence of spermatocytes at 16 dpp **(I)** and arrest of spermatogenesis at 64 dpp **(J)** with histological alterations, such as cellular degeneration and epithelial vacuolization. In the CH group, electron microscopy showed a delay in the migration of gonocytes until 10 dpp **(P–R)**, cellular degeneration, vacuolization and Sertoli cell nuclei with deep indentations (S, T and image insert T) and displaced towards the central part of the cord (S, insert). Congenital hypothyroidism (CH), Gonocytes (g), Spermatogonia (Sg), spermatocytes (Sc), round (Rs) and elongated spermatids (Es), nuclei of Sertoli cells (s), myoid cells (m), degenerating cells (d), vacuoles (v). Toluidine Blue **(A–J)**. Scale bar: 20 μm. Electron microscopy **(K–T)**. Scale bar 2 μm.

The seminiferous cords/tubes of the animals in the Control group showed a normal cytoarchitecture, in accordance to their age. At 5 dpp, the gonocytes were found in migration phase towards the basement membrane, and occasionally, in the center of the cords (Figure 2A). At 8 dpp, the first spermatogonia were observed (Figure 2B). At 10 dpp, the first spermatocytes emerged (Figure 2C) and at 16 dpp, an increase in their proliferation was observed (Figure 2D). At 64 dpp, the seminiferous epithelium showed a complete spermatogenic process from spermatogonia to elongated spermatids (Figure 2E). In the seminiferous cords/tubes of animals with CH, as compared to the Control, a greater number of gonocytes was observed at 5, 8 and 10 dpp with a decrease in the number of spermatogonia starting at 8 dpp (Table 1; Figures 2F–H *p* < 0.05); thus, generating a reduction in the gonocyte differentiation index (Table 1, *p* < 0.05) at 8 and 10 dpp. Spermatocytes were evident until 16 dpp (Figure 2I); although, they were rarely present in some cords at 10 dpp. Germ cell populations were lower at all ages (Table 2, *p* < 0.05). The population of SCs was significantly reduced (*p* < 0.05) at 8 and 10 dpp; however, at 16 and 64 dpp, these cells were present in greater numbers (Table 2, *p* < 0.05). The MI was reduced starting from 8 dpp, while the HI increased (*p* < 0.05) at all ages, characterized by desquamation, vacuolization, folding of the basement membrane and pyknosis (Table 2, Figures 2G–J). In the animals with CH at 64 dpp, spermatogenesis was arrested, and spermatogonia, spermatocytes and in some cases round spermatids were observed in the seminiferous tubes (Figure 2J).

**TABLE 2:** Testicular histological parameters in Control and CH animals.

Age (dpp)	Group	Sc/cord/tube	Rs/tube	Es/tube	S/tube	Area/cord/tube (µm^2^ X10^2^)	MI	HI
***5***	*CONTROL*	-	-	-	28.76 ± 1.90	22.85 ± 2.03	2	0.29 ± 0.12
	*CH*	-	-	-	25.80 ± 1.51	13.30 ± 1.99*	2	1.30 ± 0.46*
***8***	*CONTROL*	-	-	-	31.53 ± 0.75	31.58 ± 2.18	2.96 ± 0.05	0.36 ± 0.17
	*CH*	-	-	-	28.13 ± 2.41*	14.92 ± 8.17*	2.34 ± 0.12*	1.16 ± 0.25*
***10***	*CONTROL*	1.85 ± 0.63	-	-	39.70 ± 2.64	52.82 ± 4.27	2.76 ± 0.42	0.74 ± 0.15
	*CH*	0.65 ± 0.27*	-	-	32.60 ± 3.18*	35.44 ± 2.61*	2.28 ± 0.45*	1.88 ± 0.59*
***16***	*CONTROL*	16.73 ± 3.00	-	-	44.65 ± 2.62	68.56 ± 7.64	4.90 ± 0.37	1.25 ± 0.46
	*CH*	2.17 ± 0.73*	-	-	50.20 ± 3.86*	49.09 ± 5.93*	3.42 ± 0.70*	7.72 ± 0.60*
***64***	*CONTROL*	61.71 ± 7.71	91.75 ± 19.73	72.06 ± 9.82	26.49 ± 1.82	707.61 ± 248.90	9.85 ± 0.39	1.65 ± 0.28
	*CH*	50.55 ± 8.26	31.00 ± 12.27*	2.40 ± 1.46*	29.14 ± 3.55*	391.47 ± 53.78*	7.97 ± 0.61*	5.04 ± 0.63*

Data are expressed as mean ± SEM, of 5 animals/group. Analyzing 15 to 20 seminiferous cords/tubes per animal. Statistically significant difference compared to the Control group = *p* < 0.05. dpp: days *postpartum*, CH: congenital hypothyroidism, Sc: Spermatocytes, Rs: Round spermatids, Es: Elongated spermatids, S: sertoli cell, MI: maturation index, HI: histopathological index.

In the Control group, electron microscopy showed few cells in degeneration and with compact to slightly diffuse basement membranes at all ages, intact nuclei of the SCs moved to the base of the seminiferous epithelium from 16 dpp (Figures 2K–O). On the contrary, electron microscopy of the groups with CH showed germ cells in the process of degeneration and diffused basal membranes at all ages. SC nuclei were displaced towards the apical part of the seminiferous epithelium from 16 dpp, and these were observed with deep indentations of the nuclear envelope (Figures 2P–T).

**FIGURE 3:**
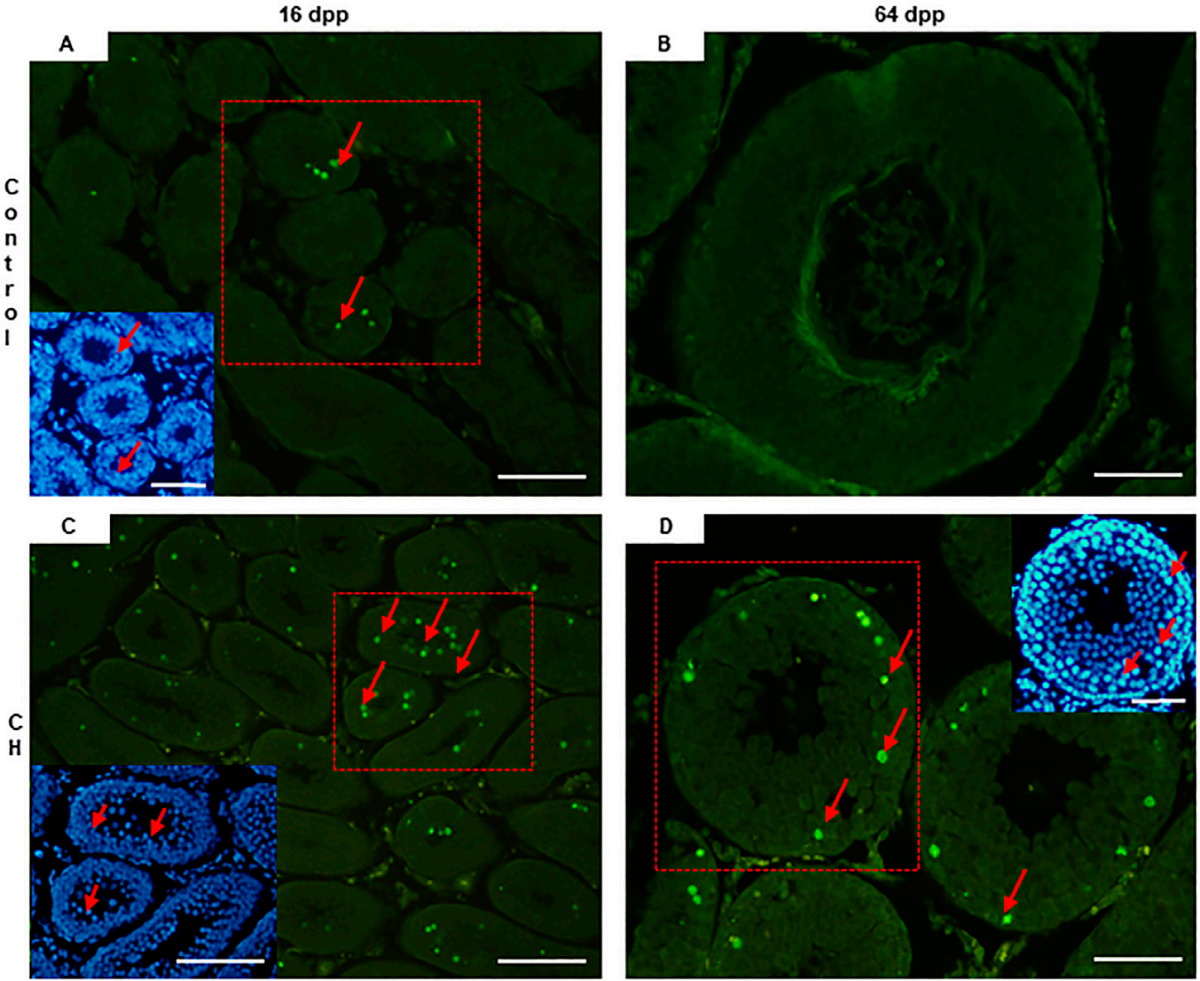
Seminiferous tubes with cells in apoptosis. **(A)** 16 dpp Control; **(C)** CH of 16 dpp; **(B)** 64 dpp Control; **(D)** CH of 64 dpp. At both ages, a greater number of apoptotic spermatocytes were seen in the CH group. Congenital hypothyroidism (CH). TUNEL. Inserts DAPI. Scale bar 20 µm.

In the CH group, apoptosis was found to be significantly increased (*p* < 0.05) at 16 and 64 dpp when compared to the Control group (Table 3, Figures 3A–D).

**TABLE 3:** Cell apoptosis and immunoreactivity.

Age (dpp)	Group	TUNEL (+) (Number/cord/tube)	Pou5f1 (+) (Number/cord)	Stra8 (+) (Number/cord/tube)	Pcna (+) (Sertoli cell/cord)	Ar (+) (Number/cord/tube)
***5***	*CONTROL*	0.16 ± 0.09	1.69 ± 0.80	-	20.88 ± 1.69	-
	*CH*	0.21 ± 0.05	1.85 ± 0.53	-	22.73 ± 1.45	-
***8***	*CONTROL*	0.26 ± 0.08	1.47 ± 0.31	9.72 ± 1.04	24.10 ± 2.11	12.76 ± 2.51
	*CH*	0.31 ± 0.09	1.65 ± 0.31	6.95 ± 0.10*	24.49 ± 1.44	1.22 ± 0.66*
***10***	*CONTROL*	0.51 ± 0.21	1.26 ± 0.09	9.9 ± 2.62	25.00 ± 0.41	21.82 ± 3.21
	*CH*	0.68 ± 0.31	1.86 ± 0.03*	3.66 ± 0.56*	24.15 ± 2.39	3.21 ± 3.06*
***16***	*CONTROL*	0.79 ± 0.30	1.31 ± 0.24	28.53 ± 1.52	34.52 ± 1.02	36.86 ± 5.41
	*CH*	3.49 ± 1.66*	1.38 ± 0.30	11.02 ± 1.81*	38.21 ± 2.26*	8.14 ± 3.27*
***64***	*CONTROL*	1.75 ± 0.19	-	59.70 ± 13.64	-	48.02 ± 3.11
	*CH*	7.14 ± 1.34*	-	34.05 ± 21.67*	-	26.59 ± 2.26*

Data are expressed as mean ± SEM, of 5 animals/group. Analyzing 15 to 20 seminiferous cords/tubes per animal. Statistically significant difference compared to the Control group = *p* < 0.05. dpp: days *postpartum*, CH: congenital hypothyroidism, Pou5f1: POU, class 5 homeobox 1, Stra8: stimulated by retinoic acid gene 8, Pcna: proliferating-cell nuclear antigen, Ar: Androgen Receptor.

Therefore, it was not possible to determine the sperm parameters in the CH group since no sperm were observed in the epididymis cauda, a situation contrary to the Control group where the sperm parameters showed a vitality of 73.2% ± 8.21%, concentration of 14.7 ± 1.19 × 10^6^/mL and normal morphology of 83.8% ± 3.96%.

#### 3.2.2 Immunohistochemical analysis and relative expression of Pou5f1 and Stra8

##### 3.2.2.1 Pou5f1, protein characteristic of gonocyte; and Stra8, protein characteristic of spermatogonia

**FIGURE 4:**
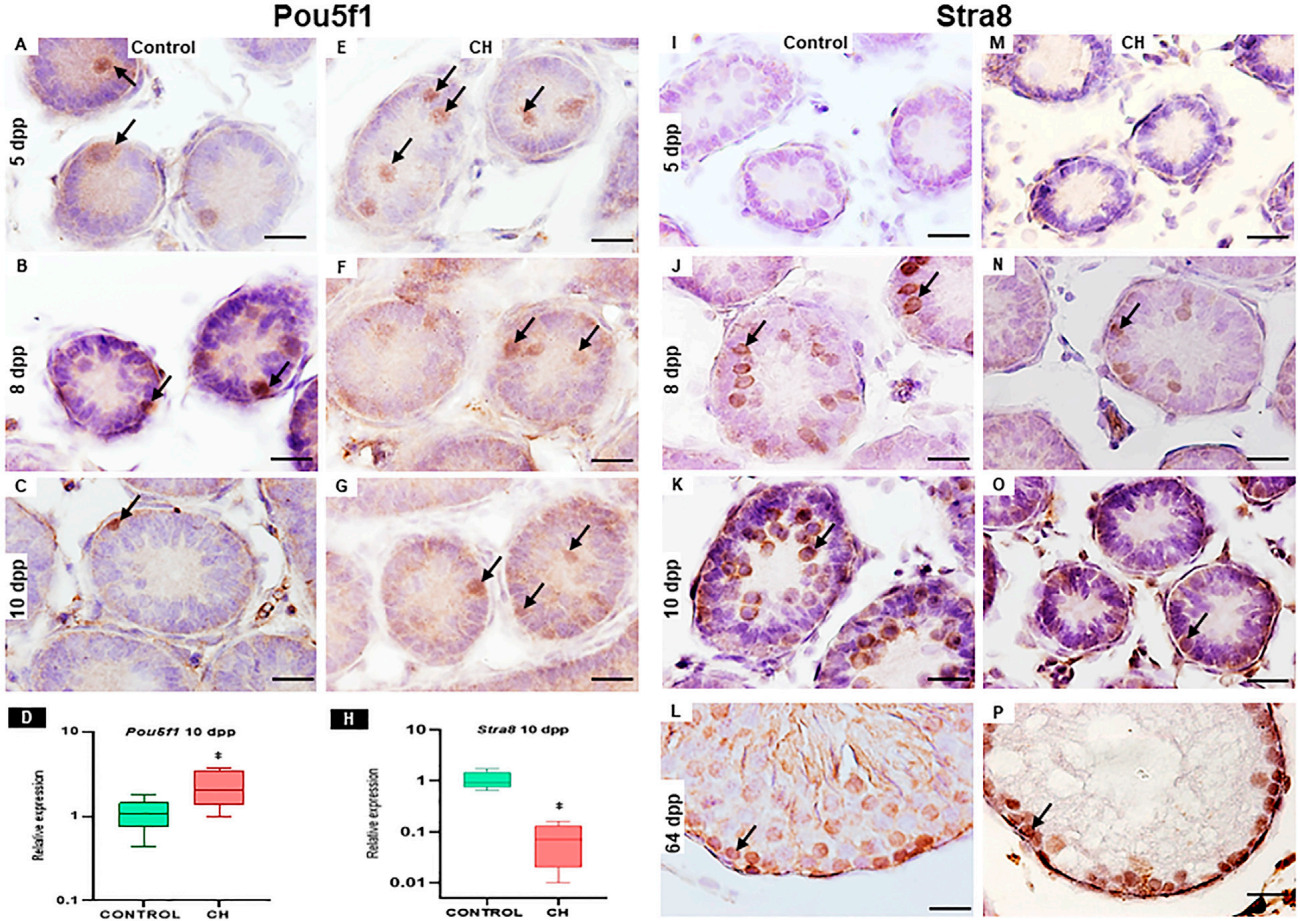
Cords and seminiferous tubes from Control and CH animals where immunoreactivity to Pou5f1 (arrow **(A–G)** and Stra8 (arrow **(I–P)**) can be seen. Greater immunoreactivity to Pou5f1 was observed at 10 dpp in the CH group (G) unlike the Control group (C). The Stra8 protein becomes evident from 8 dpp, with greater immunoreactivity in the Control group at all ages **(I–L)** compared to the CH group **(M–P)**. Figures D and H show the relative expression of Pou5f1 and Stra8 at 10 dpp. Congenital hypothyroidism (CH). Scale Bar 20 μm.

In the control group, immunoreactivity of Pou5f1 on day 5 and 8 was observed in gonocytes located in the center and periphery of the seminiferous cords and at 10 in gonocytes located at the periphery of the cords (Figures 4A–C). Immunoreactivity to Stra8, characteristic of spermatogonia, was observed from 8 dpp (Figures 4J–L). In the CH group at 5 and 8 dpp, Pou5f1 immunoreactivity was observed in gonocytes located mainly in the center of the seminiferous cords (Figures 4E, F) and at 10 dpp, unlike the Control, the number of gonocytes immunoreactive to the Pou5f1 protein increased (*p* < 0.05) (Table 3; Figure 4G). Starting from 8 dpp, the Stra8 protein was significantly reduced (*p* < 0.05, Figures 4N–P) with respect to the Control group (Table 3; Figures 4J–L).

The results of the relative expression at 10 dpp showed a significant increase in Pou5f1 (*p* < 0.05) and a significant reduction in Stra8 (*p* < 0.05) (Figures 4D, H) when compared with the Control group.

### 3.3 Role of Sertoli cells

#### 3.3.1 Pcna, vimentin and Ar (proteins that allow the determination of the functionality of Sertoli cells)

**FIGURE 5:**
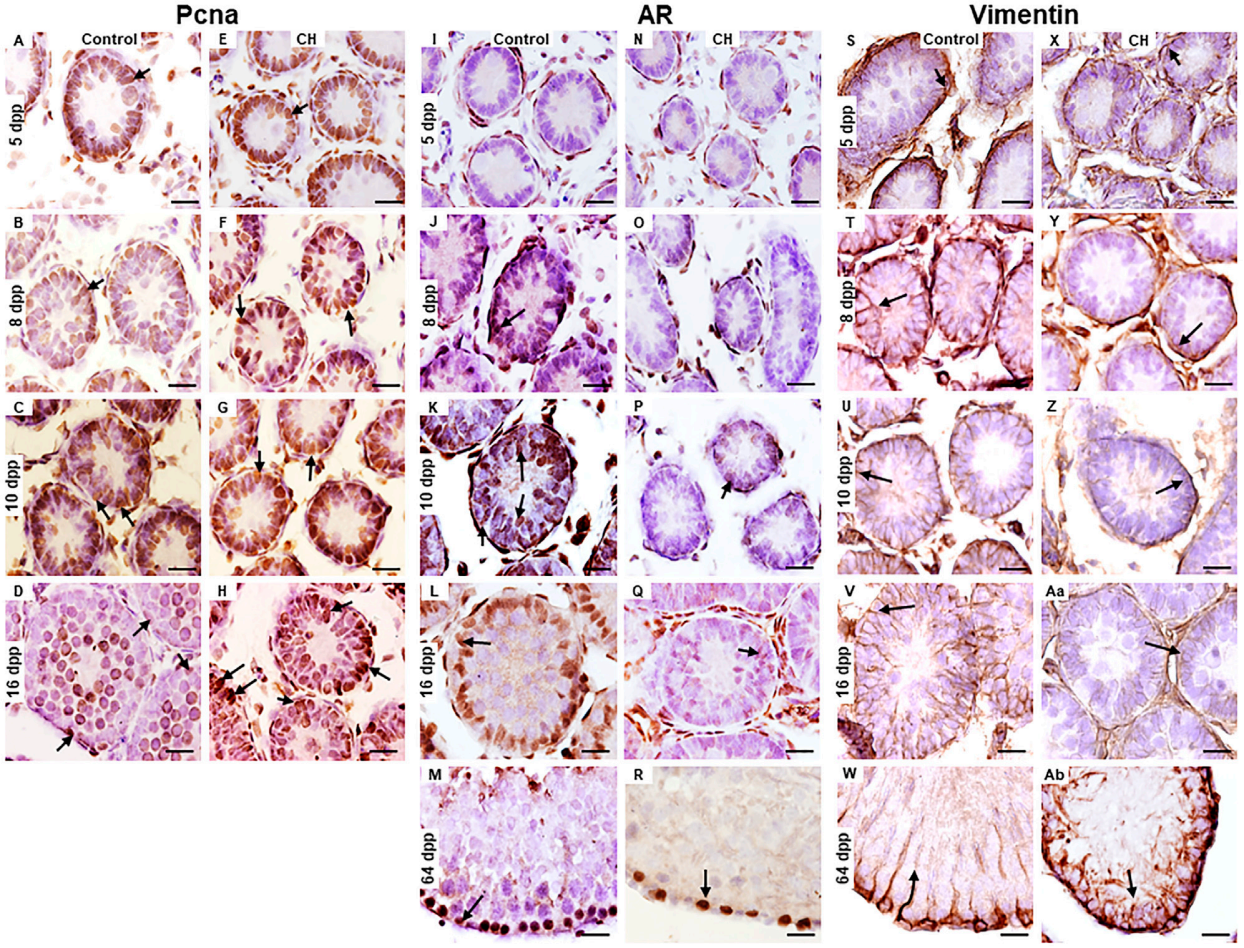
Cells in the cords and seminiferous tubes of Control and CH animals immunoreactive to the Pcna, Ar and Vimentin proteins. Sertoli cells immunoreactive for Pcna at 16 dpp were higher in the CH group (arrows E-H) unlike the Control **(A–D)**. At 64 dpp, no Pcna immunoreactivity was observed in Sertoli cells. Immunoreactivity for Ar **(I–R)** in Sertoli cells was reduced in the CH group **(N–R)** unlike the Control **(I–M)** from 8 dpp. Immunoreactivity for vimentin was greater in the Control group **(S–W)**, when compared the CH group **(X-Ab)** where its distribution was disorganized. Congenital hypothyroidism (CH). Scale bar 20 μm.

In the Control group, cell proliferation determined by Pcna immunoreactivity in SCs was observed from 5 to 16 dpp. (Figures 5A–D). The presence of Ar was evident from 8 dpp and the number of cells immunoreactive to this receptor increased reaching its maximum at 64 dpp (Figures 5J–M). The distributed of vimentin in the basal cytoplasm of the SCs took place from 5 to 10 dpp, when it was appreciated to surround the nucleus of these cells (Figures 5S–U). At 16 and 64 dpp, the distribution was perpendicular to the basal membrane; thus, facilitating the support of germ cells (Figures 5V, W). In the CH group, Pcna immunoreactivity presented a distribution pattern similar to the Control group, except at 16 dpp when it became significantly increased (*p* < 0.05) (Table 3; Figures 5E–H). The number of Ar-immunoreactive cells in the CH group was observed to reduced significantly (*p* < 0.05) from 8 dpp onwards (Table 3; Figures 5N–R) when compared with the Control group. In the groups with CH, vimentin distribution until 10 dpp was similar to that of the Control group; however, at 16 dpp, it remained concentrated at the base of the cytoplasm of the SC, which became more evident at 64 dpp when it was observed disorganized (Figures 5X–Ab).

## 4 Discussion

CH generated during gestation with MMI administration and using rdw rats with CH due to missense mutation of the thyroglobulin gene is associated with alterations in fertility of their male offspring through its negative effect on sperm quality or inhibition of spermatogenesis (Umezu et al., 2004; Anbalagan et al., 2010; Aiceles et al., 2017).

Considering that in early postnatal stages, there is a maximum expression of TRs in testicular cells (Jannini et al., 1990; Canale et al., 2001; Holsberger and Cooke, 2005), it was interesting to study how CH affected the early processes of gonocyte differentiation and meiosis of the first spermatogenic waves.

The CH model achieved by the administration of MMI during pregnancy was confirmed in our study by the low concentrations of T_4_ plus the thyrocyte hypertrophy that occurred at all ages, as well as the reduction in body and testicular weight after 16 dpp.

In the present study, a reduction in the differentiation index of gonocytes in the CH group was observed. This reduction was demonstrated by the increase in the number of gonocytes, the reduction in the number of spermatogonia and in the number of immunoreactive cells to Stra8 at 8, 10 and 16 dpp, added to an increase in the number of immunoreactive cells to Pou5f1 at 10 dpp supported by the expression of the messengers of these proteins at this same age. This event resulted in a lower population of germ cells, reduced area of the cords/tubes seminiferous with histological alterations. Furthermore, once spermatogenesis began, it did not continue beyond pachytene spermatocytes and on rare occasions, to round spermatids. This led to poor maturation of the seminiferous epithelium with azoospermia.

It has been reported that neonatal hypothyroidism delays the differentiation of gonocytes, since its population is increased, possibly due to arrest of their migration to the basement membrane (Simorangkir et al., 1997). However, this report did not point out a possible cause of this event. The administration of T_3_ in healthy neonatal rats tends to increase the differentiation of gonocytes and the number of spermatogonia (Faraone-Mennella et al., 2009; Marchlewska et al., 2013; Marchlewska et al., 2011). This may have resulted from the increase in platelet-derived growth factor beta receptor (PDGFR-β) protein, which is known to be involved in gonocyte differentiation (Basciani et al., 2008; Basciani et al., 2010). T_3_ treatment increases PDGFR-β levels in the heart of mice with PTU-induced hypothyroidism (Chen et al., 2012).

In line with the above, other authors have also reported a delay or an arrest of spermatogenesis in spermatocyte or spermatid using different CH models (Francavilla et al., 1991; Sakai et al., 2004; Yildirim et al., 2017). Furthermore, the presence of TRα has been demonstrated in intermediate spermatogonia and even in pachytene spermatocytes, which coincides with the low amount of these cell types due to the absence of the hormone (Jannini et al., 1999; Buzzard et al., 2000). Sarkar and Singh (2017) mentioned that TRα1 immunostaining is intense in the acrosomal region of elongated spermatids and when the neonatal hypothyroidism model with PTU is developed, it leads to the absence of elongated spermatids. This demonstrates the importance of THs in cell differentiation during spermiogenesis.

Retinoic acid is known to play an important role in gonocyte differentiation and the initiation of meiosis, inducing the expression of the Stra8 protein in A1 spermatogonia, preleptotene and pachytene spermatocytes (Koubova et al., 2006; Green et al., 2018). The presence of retinoic acid receptors in Sertoli and germ cells has been demonstrated, and their alteration generates fertility failures (Griswold, 2016). CH has been documented to affect the expression of retinoic acid receptor β in the liver (Pallet et al., 1994). It is likely that the deficiency of this receptor is related to the decrease in the immunoreactivity to the Stra8 protein and its expression at 10 dpp, which alters the differentiation of gonocytes and the continuation of meiosis. This suggests the possibility of Sertoli cell dysfunction, without ruling out that CH may directly affect post-gonocyte germ cells, since these cell types also present TRs (Buzzard et al., 2000; Fadlalla et al., 2017). TRs in germ cells have been associated with their survival (Buzzard et al., 2000). CH generates a reduction or absence in the expression of TRs (Fadlalla et al., 2017; Sarkar and Singh, 2017). This probably led to apoptosis of testicular cells as seen in this work.

In the present study, it was observed that from 8 dpp through all the ages, the number of SCs immunoreactive to Ar in animals with CH was lower. It has been documented that T_3_ upregulates the mRNA for Ar in SCs (Arambepola et al., 1998) since this steroid is important to support their differentiation and survival (Walczak-Jedrzejowska et al., 2007). Panno et al. (1996) mentioned that T_3_ increases Ar in peripubertal SCs. When T_3_ and androgens are administered together, they generate a positive regulation of Ar, which could support the role of both hormones in the responsiveness of SCs to androgens during spermatogenesis (Panno et al., 1996). It was reported that Ar deficiency is associated to immaturity of SCs (Arambepola et al., 1998). This immaturity has also been demonstrated in neonatal hypothyroidism due to estrogen production, characteristic of immature SCs (Sarkar and Singh, 2017). In addition, this situation can be accentuated by the continued proliferation of SCs beyond 15 dpp (Gaytan et al., 1986; Picut et al., 2015), that brought about an increase in the population of these cells, as was observed in the present work at 16 dpp in the CH group. Therefore, these facts support the theory of a delay in maturation or dysfunction of SCs that affects the synthesis and/or transmission of molecules necessary for the maintenance and differentiation of germ cells.

Alterations in the transmission of maintenance and differentiation molecules may be due to the effects on connexin 43, which has been shown to be regulated by THs (Gilleron et al., 2006; Rodrigues et al., 2022). The expression of this connexin is reduced in PTU induced neonatal hypothyroidism (Gilleron et al., 2006). Connexin 43 is a component of gap junctions that allows communication among SCs, and between these cells and germ cells (Decrouy et al., 2004); thus, it helps to maintain the ultrastructure and maturation of SCs. The seminiferous epithelium of Sertoli cell-specific connexin 43 knockout mice (SCCx43KO^−/−^) has been reported to show vacuolization caused by the absence of germ cells with disorganization of the cytoplasmic processes of SCs. These cells lose polarity, generating nuclei with deep indentations of their nuclear envelope associated with vimentin and their displacement towards the central region of the tubes (Aumüller et al., 1992; Staggenborg et al., 2022), as observed in the present work on animals with CH. The gonocytes, the spermatogonia and the spermatocytes that failed to receive molecules for their maintenance and differentiation degenerated by apoptosis as observed at 16 and 64 dpp, which generated a reduction in the population of germ cells and the absence of spermatozoa. A reduction in the number of germ cells and live sperm has been reported in rats with persistent hypothyroidism from birth (Sahoo et al., 2008; La Vignera and Vita, 2018). It is mentioned that this reduction may be caused by an imbalance between the increase in free radicals and the reduction of the endogenous antioxidant system, such as catalase and superoxide dismutase (Choudhury et al., 2003; Sahoo et al., 2008). In addition, Zamoner et al. (2008) reported that superoxide dismutase, glutathione S-transferase and Glutathione reductase enzymes were affected by PTU-induced CH. In this work, however, the endogenous antioxidant system enzymes were not determined.

Furthermore, alterations in vimentin distribution in the CH group backs up the idea of Sertoli cell dysfunction. Vimentin is an intermediate microfilament that is expressed in SCs (Romeo et al., 1995; Show et al., 2003; Franke et al., 2004). It plays several roles in cell architecture, such as maintaining the cytoplasmic structural integrity that gives support to developing germ cells, surrounding the nucleus and keeping it fixed in its location, being deposited in the indentations of the nuclear envelope, and forming binding structures that are necessary for the transmission of molecules for spermatogenesis (Russell and Peterson, 1985; Aumüller et al., 1992; Tanemura et al., 1994; Show et al., 2003). Zamoner et al. (2008) reported that CH generates hyperphosphorylation of vimentin without affecting its expression**,** but leads to accumulation of insoluble vimentin. Cell junction and communication proteins are important for the migration of gonocytes from the center of the cord to the basal membrane and for the movement of spermatocytes from the basal to the apical compartment, as well as for the transmission of molecules from the SCs for their maintenance and differentiation. Hence, the disorganization of vimentin, as observed in this study, could have interfered with the migration of gonocytes and their differentiation to spermatogonia in the CH rat model.

It would be important to analyze whether the administration of T_4_ in CH model, favors the functioning of Sertoli cells by generating differentiation of gonocytes, completion of meiosis and conclusion of spermiogenesis, reducing cell death and restoring sperm parameters.

## 5 Conclusion

CH generated a delay in the differentiation of gonocytes, arrest of meiosis and conclusion of spermiogenesis. This may be caused by alterations in the SCs functions as was demonstrated by the deficiency in immunoreactivity to Ar, vimentin and the presence of altered nuclei with deep indentations that probably generated a deficiency in the synthesis and/or transmission of proteins and factors necessary for differentiation, which could have had an impact on poor cell proliferation and differentiation with sperm absence.

## Data Availability

The raw data supporting the conclusion of this article will be made available by the authors, without undue reservation.
