# Supplementary figures and images for: Methimazole-induced congenital hypothyroidism affects gonocytes differentiation and arrests meiosis: role of Sertoli cells
Source: Front Cell Dev Biol. 2024 Nov 21;12:1493872. doi: 10.3389/fcell.2024.1493872 (PMC11617512; doi:10.3389/fcell.2024.1493872)

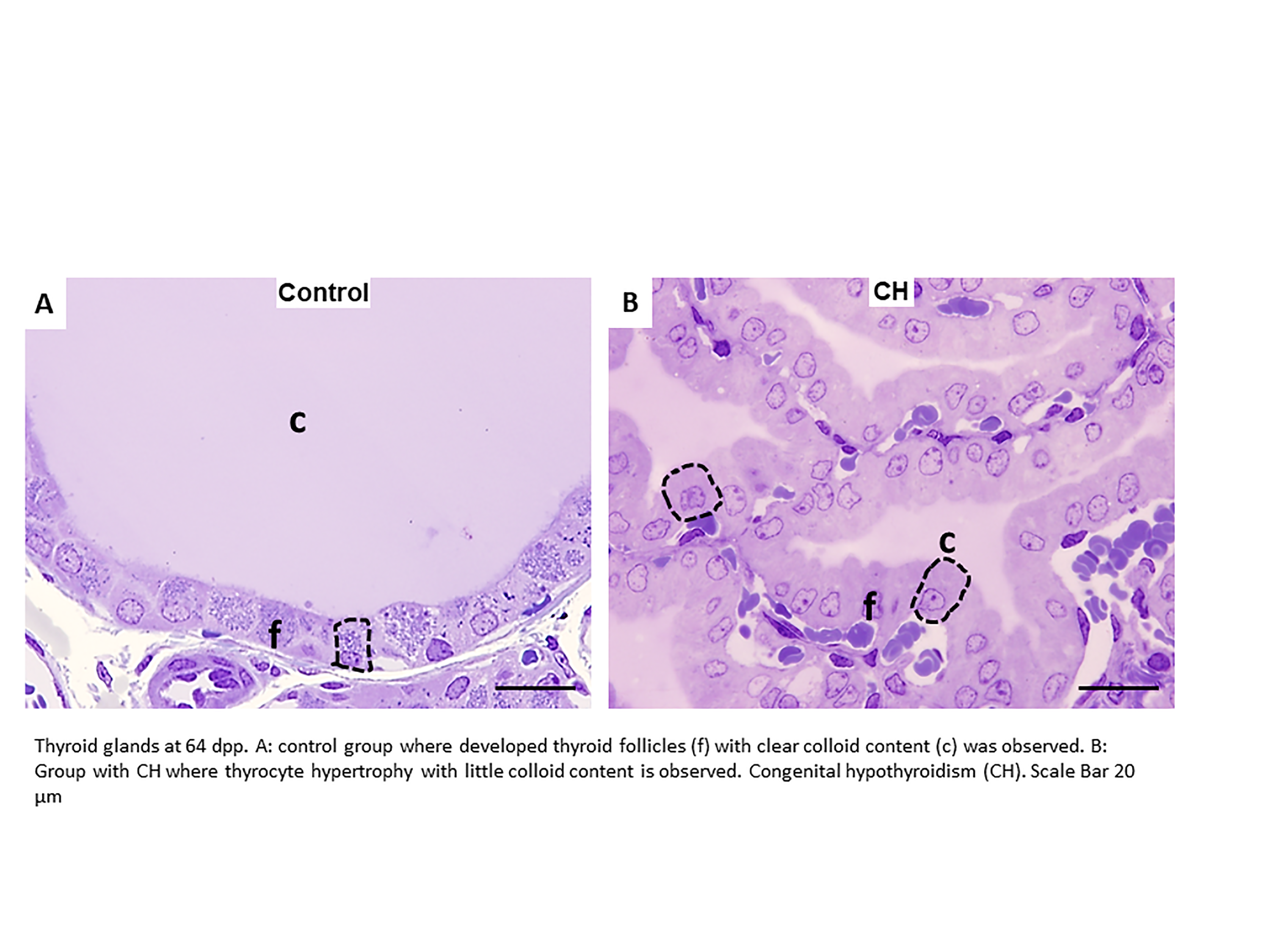

Supplement: Supplementary file 1 [file Image1.tif]
